# Anti-tumor Activity and Epigenetic Impact of the Polyphenol Oleacein in Multiple Myeloma

**DOI:** 10.3390/cancers11070990

**Published:** 2019-07-16

**Authors:** Giada Juli, Manuela Oliverio, Dina Bellizzi, Maria Eugenia Gallo Cantafio, Katia Grillone, Giuseppe Passarino, Carmela Colica, Monica Nardi, Marco Rossi, Antonio Procopio, Pierosandro Tagliaferri, Pierfrancesco Tassone, Nicola Amodio

**Affiliations:** 1 Department of Experimental and Clinical Medicine, *Magna Graecia* University of Catanzaro, 88100 Catanzaro, Italy; 2 Department of Health Science, *Magna Graecia* University of Catanzaro, 88100 Catanzaro, Italy; 3 Department of Biology, Ecology and Earth Sciences (DiBEST), University of Calabria, 87036 Arcavacata di Rende, Italy; 4 CNR, IBFM UOS of Germaneto, *Magna Graecia* University of Catanzaro, 88100, Catanzaro Italy

**Keywords:** experimental therapeutics, HDAC, multiple myeloma, oleacein

## Abstract

Olive oil contains different biologically active polyphenols, among which oleacein, the most abundant secoiridoid, has recently emerged for its beneficial properties in various disease contexts. By using in vitro models of human multiple myeloma (MM), we here investigated the anti-tumor potential of oleacein and the underlying bio-molecular sequelae. Within a low micromolar range, oleacein reduced the viability of MM primary samples and cell lines even in the presence of bone marrow stromal cells (BMSCs), while sparing healthy peripheral blood mononuclear cells. We also demonstrated that oleacein inhibited MM cell clonogenicity, prompted cell cycle blockade and triggered apoptosis. We evaluated the epigenetic impact of oleacein on MM cells, and observed dose-dependent accumulation of both acetylated histones and α-tubulin, along with down-regulation of several class I/II histone deacetylases (HDACs) both at the mRNA and protein level, providing evidence of the HDAC inhibitory activity of this compound; conversely, no effect on global DNA methylation was found. Mechanistically, HDACs inhibition by oleacein was associated with down-regulation of Sp1, the major transactivator of HDACs promoter, *via* Caspase 8 activation. Of potential translational significance, oleacein synergistically enhanced the in vitro anti-MM activity of the proteasome inhibitor carfilzomib. Altogether, these results indicate that oleacein is endowed with HDAC inhibitory properties, which associate with significant anti-MM activity both as single agent or in combination with carfilzomib. These findings may pave the way to novel potential anti-MM epi-therapeutic approaches based on natural agents.

## 1. Introduction

Multiple myeloma (MM) is a clonal B cell malignancy characterized by the accumulation of tumor plasma cells (PCs) in the bone marrow (BM), where different cell types establish a complex microenvironment that supports survival, proliferation and drug-resistance of the malignant clone. The last few years have witnessed a rapid development of drugs for the treatment of this malignancy, leading to increased extent and frequency of response and to the improvement in median overall survival of patients. However, despite such therapeutic advancements, MM eventually evolves into a drug-resistant phase leading to patients’ death [1]. This finding has stimulated continuous investigation on new therapeutic options, as single agents or in combination with established anti-MM drugs. In this regard, natural compounds have recently emerged as novel chemopreventive and/or therapeutic tools able to target oncogenic pathways involved in the pathogenesis of human malignancies. Significant anti-inflammatory, anti-oxidant and cytotoxic effects of natural agents have been demonstrated also in MM, either by epidemiologic or animal studies, and even by clinical trials [2]. Several natural compounds from various plants, fungi and marine organisms have been shown to target epigenetic events underpinning tumorigenesis, such as DNA methylation, histone modifications (methylation, acetylation and phosphorylation), and non-coding RNAs [3], known to be deeply dysregulated and representing valuable therapeutic targets in MM [2].

Polyphenols are important constituents of several plants and vegetables, recognized as powerful anti-oxidants endowed with anti-inflammatory, antimicrobial and antitumor activities [4]. Indeed, a major source of polyphenols is represented by extra virgin olive oil (EVOO), whose polyphenolic fraction includes simple phenols (tyrosol and hydroxytyrosol), secoiridoids (oleuropein, oleocanthal and oleacein), and lignans. EVOO-derived secoiridoids, characterized by the presence of elenoic acid or its derivatives in their molecular structure, have been shown to prevent obesity, osteoporosis, and neurodegeneration. Oleocanthal has shown anti-tumor activity in different types of tumors, including MM, hepatocellular carcinoma, breast, prostate, pancreatic cancers and melanoma [5,6,7,8,9]. The most abundant secoiridoid of EVOO is the dialdehydic form of elenolic acid conjugated with 3,4-(dihydroxyphenyl)ethanol (3,4-DHPEA-EDA), also known as oleacein, whose anti-oxidant, anti-inflammatory, and anti-microbial properties have recently emerged [10], while its effects on tumor biology are still poorly defined.

We here aimed to investigate the anti-tumor potential of oleacein against MM. Our results highlight a previously unknown epigenetic impact of oleacein on MM cells, with potential implications for the management of MM and possibly other malignancies.

## 2. Results

### 2.1. Inhibitory Effects of Oleacein on MM Cell Viability and Survival

**Figure 1:**
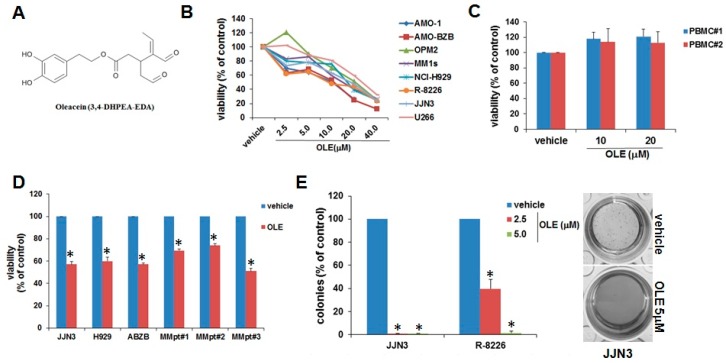
Effects of oleacein on multiple myeloma (MM) cell survival. (**A**) Chemical structure of oleacein. (**B**) Cell viability of MM cell lines as determined by Cell Titer Glo (CTG) assay 48 h after treatment with increasing doses of oleacein or vehicle (DMSO). (**C**) CTG assay performed on peripheral blood mononuclear cells (PBMCs) from three different healthy donors treated with oleacein for 48 h. (**D**) CTG assay in MM cell lines and primary CD138^+^ cells from three MM patients (MM pt#1, #2 and#3) co-cultured on HS-5 stromal cells and treated for 48 h with 5.0μM oleacein. (**E**) Colony formation assay performed on MM cell lines treated for 14 days with oleacein; representative pictures of JJN3 colonies at day 14 are shown in the right panel (5× magnification). * *p* < 0.05 as compared to vehicle-treated cells.

Oleacein, whose chemical structure is reported in Figure 1A, was obtained by a green semi-synthetic modification of oleuropein as previously reported [11]. By using a panel of eight different MM cell lines carrying the major cytogenetic aberrations of MM, we sought to analyze the impact of oleacein on cell viability. MM cells were exposed to increasing doses of oleacein, and cell viability was assessed by Cell Titer Glo (CTG) assay. Noteworthy, a dose-dependent inhibition of cell viability was observed 48 h after oleacein treatment, with IC50s ranging from 5.0 to 20.0 µM (Figure 1B); conversely, oleacein did not affect the viability of PBMCs from healthy donors (Figure 1C), suggesting a favorable therapeutic index. We next evaluated the effects of oleacein on MM cells in the presence of the BM *milieu*, which is known to trigger drug-resistance [1]. Importantly, the inhibitory effect of oleacein was maintained even when MM cell lines, or primary CD138^+^ cells purified from MM patients, were cultured in the presence of HS-5 stromal cells, thus suggesting that oleacein can overcome BM microenvironment-mediated pro-survival effects (Figure 1D). In addition, oleacein drastically suppressed the clonogenicity of MM cells in methylcellulose cultures (Figure 1E). Collectively, these data unveil an inhibitory activity of oleacein on MM cell viability and survival.

### 2.2. Oleacein Triggers Cell Cycle Arrest and Apoptosis 

**Figure 2:**
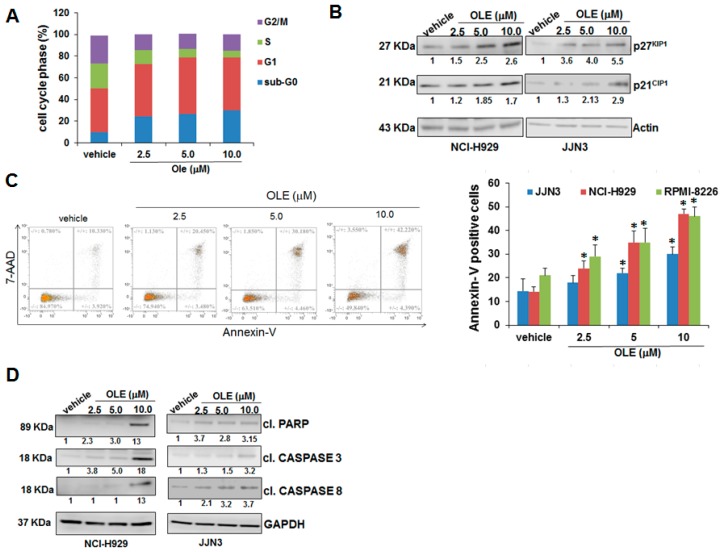
Oleacein triggers cell cycle blockade and apoptosis. (**A**) Cell cycle analysis was performed on NCI-H929 cells by PI staining, 24 h after treatment with oleacein or vehicle (DMSO). (**B**)Western Blot (WB) analysis of p27^KIP1^ and p21^CIP1^ in whole cell lysates from MM cells after treatment with oleacein for 24 h; actin was used as loading control. (**C**) Annexin V/7-AAD staining of MM cells after treatment with oleacein for 48 h; a representative experiment on NCI-H929 cells is shown on the left side. (**D**) WB of PARP1, cleaved caspase-3 and cleaved caspase-8 in NCI-H929 and JJN3 cell lines after 24 h of oleacein treatment; GAPDH was used as loading control. * *p* < 0.05 as compared to vehicle-treated cells.

To unravel the biological sequelae of oleacein in MM, we first analyzed by flow cytometry the cell cycle profile of oleacein-treated cells after propidium iodide staining. As shown in Figure 2A, oleacein increased the percentage of hypodiploid cells (sub-G0 phase), and also induced the accumulation of cells in the G0/G1 phase; WB analysis showed a dose-dependent increase of cell cycle inhibitors p27^KIP1^ and p21^CIP1^ protein expression (Figure 2B), strengthening the capability of oleacein to trigger cell cycle blockade.

In order to confirm apoptosis induction, we performed Annexin V/7-AAD staining on MM cell lines after oleacein treatment. We found an increase in late apoptotic events, which ranged from 20 to 30% after treatment with oleacein 5.0 and 10.0 µM, respectively (Figure 2C); the increase in cleaved PARP1, caspase-3 and caspase-8 on oleacein-treated MM cell lines, as shown by Western Blot (WB), further confirmed apoptosis induction (Figure 2D); no activation of caspase-7 and -9 was observed (Supplementary Figure S1), thus indicating that oleacein predominantly activates the extrinsic apoptotic pathway. These results therefore indicate that oleacein may elicit anti-MM activity through modulation of cell cycle and apoptosis.

### 2.3. HDAC Inhibitory Activity of Oleacein in MM

**Figure 3:**
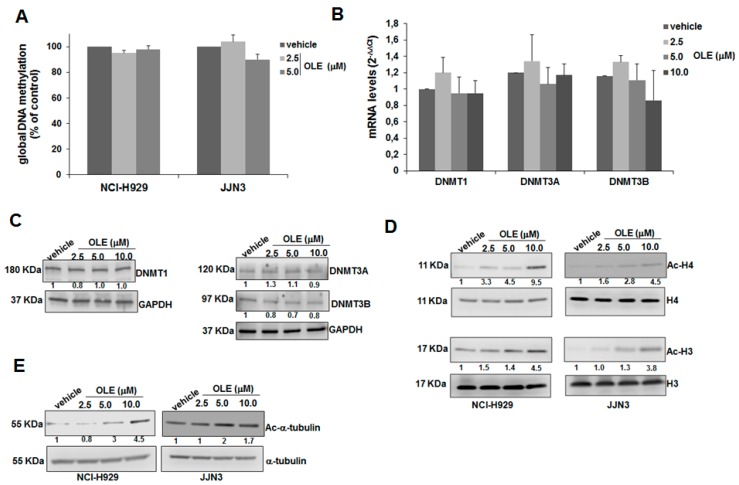
Oleacein affects the acetylome but not the methylome of MM cells. (**A**) Global DNA methylation was measured in MM cells treated for 24 h with oleacein, as reported in materials and methods. Quantitative Real Time PCR (QRT-PCR) (**B**) and WB analysis (**C**) of DNMT1, DNMT3A and DNMT3B in JJN3 cells treated for 24 h with oleacein; GAPDH was used as loading control. WB analysis of acetylated histone H3, histone H3, acetylated histone H4, histone H4 (**D**) and acetylated α-tubulin (**E**) in NCI-H929 and JJN3 cells treated with oleacein for 24 h; GAPDH was used as loading control.

Aberrant epigenetic patterns are common in MM, where they are frequently associated with disease onset and/or progression to advanced stages [12,13,14]. A large body of literature has highlighted the capability of several natural compounds to revert the altered epigenome of MM cells by counteracting key oncogenic epigenetic regulators [2]. On this basis, we investigated the epigenetic impact of oleacein on MM cells, by analyzing its effects both on global DNA methylation (GDM) and histone acetylation, the two major epigenetic mechanisms dysregulated in MM. Oleacein did not significantly modify the whole content of methylated cytosines in DNA from NCI-H929 and JJN3 cell lines (Figure 3A); in line with the latter finding, no significant changes in mRNA or protein levels of DNA methyltransferases (DNMT1, DNMT3A and DNMT3B) were observed upon oleacein treatment, as shown by QRT-PCR (Figure 3B) and WB analyses (Figure 3C). Conversely, oleacein induced a significant increase in acetylated histone H3, histone H4 (Figure 3D) and α-tubulin (Figure 3E). Collectively, these findings suggest that oleacein is able to modulate the acetylome of MM cells.

**Figure 4:**
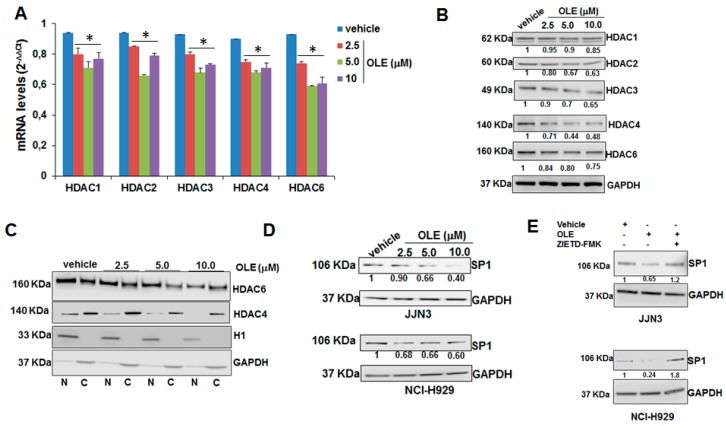
Oleacein targets HDACs. QRT-PCR (**A**) and WB analysis (**B**) of HDAC1, HDAC2, HDAC3, HDAC4, HDAC6 in JJN3 cells treated with oleacein for 24 h; GAPDH was used as loading control. (**C**) WB analysis of HDAC4 and HDAC6 in nuclear (N) and cytoplasmic (C) protein fractions from JJN3 cells treated for 24 h with oleacein; histone H1 and GAPDH were used as nuclear and cytoplasmic marker, respectively. (**D**) WB analysis of Sp1 in JJN3 cells treated with oleacein for 24 h. (**E**) WB analysis of Sp1 in JJN3 cells treated with 5.0 µM oleacein with or without 20.0 µM Z-ITED-FMK; GAPDH was used as loading control. * *p* < 0.05 as compared to vehicle-treated cells.

Aberrant expression and/or activity of HDACs drive malignant transformation of tumor cells, thus making HDACs valuable therapeutic targets in MM [13,15]. We analyzed, in oleacein-treated JJN3 cells, the mRNA and protein expression of HDACs with established oncogenic role in MM. Intriguingly, oleacein induced down-regulation of several class I/II HDACs, namely HDAC1/2/3/4/6, both at mRNA (Figure 4A) and protein level (Figure 4B); moreover, biochemical fractionation experiments indicated that oleacein reduced both the nuclear and the cytoplasmic fraction of class II HDAC4 and HDAC6, which are known to shuttle between the nucleus and the cytoplasm (Figure 4C). To understand whether oleacein could act as a canonic HDAC inhibitor, we carried out an in vitro HDAC activity assay using JJN3 nuclear extracts. Incubation with oleacein did not induce any change in the HDAC activity recovered from nuclear extracts, differently from trichostatin A (TSA) or SAHA (Supplementary Figure S2), that were used as positive controls. This finding suggests that the impact of oleacein on the acetylome of MM cells does not occur via enzymatic HDAC inhibition. We therefore explored additional mechanisms accounting for oleacein effects on HDACs, and hypothesized that oleacein could transcriptionally regulate HDACs *via* Sp1. In fact, Sp1, a ubiquitous transcription factor endowed with oncogenic activity in hematologic and solid malignancies [12,13,16,17], was proven to act as a transcriptional activator of HDACs [18]. Interestingly, oleacein treatment induced down-regulation of Sp1 (Figure 4D), and this effect occurred in a caspase 8-dependent fashion, since it was abrogated by Z-IETD-FMK, a selective caspase 8 inhibitor (Figure 4E). These results indicate that oleacein effects on HDACs expression might be mediated by Sp1.

Sp1 is involved in negative feedback loops with miRNAs, like miR-29b [19,20,21] and miR-22 [22], both acting as tumor suppressors in MM [23,24]. As expected, oleacein-induced Sp1 inhibition was paralleled by the upregulation of miR-29b and miR-22 (Supplementary Figure S3), thus strengthening the role of Sp1 pathway’s inhibition in the anti-MM activity of oleacein.

### 2.4. Oleacein Enhances the Anti-MM Activity of Carfilzomib

**Figure 5:**
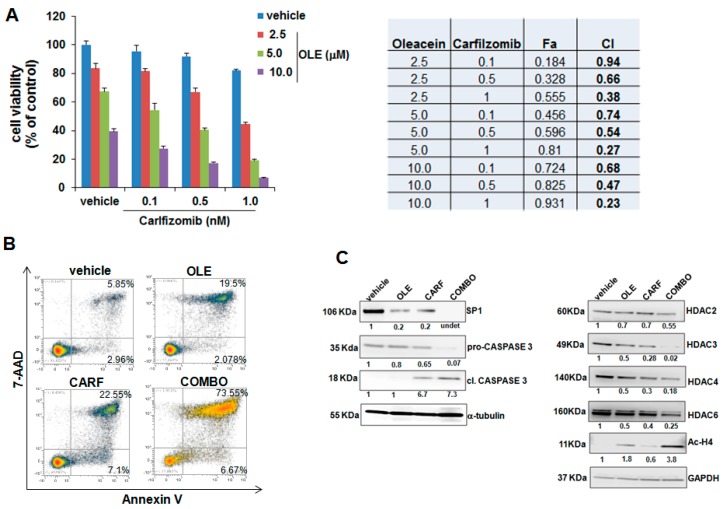
Oleacein enhances the anti-MM activity of carfilzomib. (**A**) CTG assay was performed on NCI-H929 cells treated with oleacein (2.5, 5.0 or 10.0 µM) and carfilzomib (0.1, 0.5 and 1.0 nM). Results are expressed as percentage of the viability of vehicle-treated cells. The right panel reports values of fraction affected (Fa) and combination indexes (CI) in a triplicate experiment, as calculated by the Calcusyn software. (**B**) Annexin V/7-AAD staining of NCI-H929 cells after treatment with vehicle (DMSO), 5.0 µM oleacein and 1.0 nM carfilzomib for 24 h; a representative FACS experiment is reported. (**C**). WB analysis of pro-Caspase 3, cleaved caspase 3, SP1, HDAC2, HDAC3, HDAC4, HDAC6, and acetylated histone H4 in NCI-H929 cells treated with carfilzomib (1.0 nM), oleacein (5.0 µM) or a combination of the two; α-tubulin or GAPDH were used as loading controls.

HDAC inhibitors (HDACi) are part of the therapeutic armamentarium against MM, and clinical studies have shown promising therapeutic activity of pan- or selective-HDACi when used within combination regimens [13]. On this basis, we investigated whether, similarly to pan-HDACi, oleacein treatment could trigger synergistic anti-MM activity in combination with clinically-relevant proteasome inhibitors. With this aim, NCI-H929 cells were treated with different concentrations of oleacein with or without bortezomib or carfilzomib, and subsequently cell viability was analyzed by CTG; the occurrence of synergism was assessed by Calcusyn. Interestingly, oleacein synergistically enhanced the effects of carfilzomib (CI < 1.0) on the inhibition of cell viability (Figure 5A), while combination with bortezomib was generally antagonistic (CI > 1.0; Supplementary Figure S4). Annexin V-7AAD staining of NCI-H929-treated cells indicated a higher apoptotic rate when oleacein was combined with carfilzomib, as compared to single agent treatment (Figure 5B). Accordingly, WB analysis showed increased cleavage of caspase 3 and superior Sp1 downregulation upon oleacein *plus* carfilzomib combination, thus confirming enhancement of apoptosis; moreover, oleacein *plus* carfilzomib enhanced downregulation of HDAC2, HDAC3, HDAC4 and HDAC6, with respect to single-agent treatment, which associated with increased histone H4 acetylation (Figure 5C). Collectively, these results indicate that the combination of olecein with carfilzomib results in significant acetylome derangement and apoptosis triggering of MM cells.

## 3. Discussion

Naturally occurring compounds endowed with anti-tumor activity have been found in different sources, such as vegetables, fruits, herbs and fermented products. These agents may act either by preventing the onset of primary cancer, or by antagonizing the evolution of pre-malignant and malignant lesions towards more aggressive stages. Noteworthy, experimental findings on a variety of natural compounds, including curcumin, resveratrol, celastrol and many others, have demonstrated significant advantages for the management of MM [2]. The molecular mechanisms underlying the anti-tumor activity of such compounds are diverse and only partially understood, with inhibition of oncogenic signal transduction pathways and modulation of the cellular epigenome being the most well documented. Regarding the epigenetic-modulating effects, it has been demonstrated that several natural agents, by targeting DNMTs [25], HDACs [26] or non-coding-RNAs [27], may revert aberrant epigenetic patterns implicated in the pathogenesis of human neoplasias, including MM [2].

Polyphenols found in the EVOO, a major component of mediterranean diet, have demonstrated to be protective against several diseases, including those of cardiovascular and metabolic origin [28]. The pro-active ingredient oleuropein and its derivative hydroxytyrosol have been widely studied, demonstrating many beneficial effects, both in vitro and in vivo, in experimental preclinical models [29]. Moreover, many studies have disclosed remarkable anti-tumor activity of oleuropein and hydroxytyrosol against several types of cancers [30]. However, the amount of oleuropein in the EVOO is too low to fully explain the beneficial effects deriving from EVOO assumption with diet. In fact, the endogenous β-glucosidase released during the olive oil extraction process hydrolyzes oleuropein, generating a series of degradation products, all of which are less hydrophilic than the original natural secoiridoids, and therefore more soluble in the oily matrix extracted from the drupes. Thus, EVOO is scarce in oleuropein and much more abundant in its degradation product oleacein [31], thus making it one of the most plausible effectors of the biological activity of EVOO [11,32].

Oleacein can be obtained by a simple and environmentally friendly method, starting from the easily available natural oleuropein [11]. Recent preclinical studies have highlighted anti-microbial [33], anti-inflammatory [34], and protective effects of oleacein against diet-dependent metabolic alterations [35]; conversely, its anti-antitumor activity remains poorly characterized. We here provided the first evidence of the anti-tumor activity of oleacein against MM cells: importantly, oleacein triggered cell cycle arrest and apoptosis and reduced clonogenicity, without exerting any toxic effect on healthy PBMCs, thus suggesting a favorable therapeutic index of this agent. Moreover, oleacein cytotoxic effects were also observed against primary MM cells co-cultured with BM-derived stromal cells, demonstrating the capability of oleacein to overcome BM microenvironment-dependent drug resistance.

We sought to shed light on the molecular mechanisms underlying oleacein anti-tumor activity in MM. To this aim, we focused on epigenetic mechanisms, known to be a major target of several natural agents, and whose dysregulation has been largely implicated in the pathogenesis of MM [14]. Indeed, aberrant expression of effectors of the epigenetic machinery, including DNMTs [36], HDACs [13], polycomb genes [37,38] and non-coding RNAs [39,40], has been reported in MM and has been harnessed in the context of novel anti-tumor strategies [41].

Collectively, our data indicate a strong increase in acetylation of histones and of α-acetyl-tubulin upon oleacein treatment, while no effect on GDM could be observed. This finding highlights a novel HDAC inhibitory activity of oleacein in MM. We attempted to characterize the mechanisms underlying such HDAC inhibitory effects, and found out that oleacein could transcriptionally inhibit HDACs expression likely *via* targeting of Sp1, a known transactivator of HDACs’ promoter. Since previous findings indicated that bortezomib-evoked transcriptional repression of HDACs by Sp1 occurs in a caspase 8-dependent fashion [18], we investigated whether oleacein effect on Sp1 could be similarly mediated by caspase 8. In agreement with this hypothesis, oleacein-induced Sp1 down-regulation was abrogated by the caspase 8 inhibitor Z-IETD-FMK.

Dysregulated transcription factors may drive down-regulation of tumor suppressor miRNAs in MM [12,40,42]. By establishing molecular feedback loops, Sp1, a pleiotropic transcription factor endowed with oncogenic activity in human malignancies [17], was shown to negatively affect the expression of miRNAs [40]. We have previously reported that miRNA dysregulation features prominently in the pathobiology of MM, with certain miRNAs, such as miR-125a-5p [43], miR-21 [44], miR-221 [45] and miR-17-92 cluster [46], highly expressed in MM and acting as oncogenes, while others such as miR-29b [20], miR-22 [24] and miR-125b [47], behave as tumor suppressors.

Consistent with inhibition of Sp1, oleacein triggered upregulation of tumor suppressive miRNAs, namely miR-29b [20] and miR-22 [24], which are known to be negatively regulated by this transcription factor [19,22]. These findings underscore the ability of oleacein to trigger a tumor suppressive miRNA network likely contributing to its cytotoxicity against MM cells.

Non-selective HDAC inhibitors, such as romidepsin, vorinostat and panobinostat, have shown a remarkable anti-MM effect in preclinical and clinical studies, with significant efficacy, along with reduced side effects, when given within combination regimens [13]; amongst pan-HDACi, panobinostat has been approved by FDA for MM treatment [48]. Having demonstrated its pan-HDAC inhibitory activity, and taking into account the promising clinical data which emerged from MM patients treated with proteasome inhibitors and pan-HDACi combination therapies, we also explored whether oleacein could enhance the anti-tumor activity of bortezomib or carfilzomib. Notably, when combined with carfilzomib, oleacein synergistically enhanced its in vitro cytotoxicity, with superior Sp1 and HDACs down-regulation and a resultant increase in apoptosis of MM cells. A follow-up investigation is planned to evaluate the anti-tumor effect of oleacein-based treatments in the context of validated in vivo preclinical models of human MM.

## 4. Materials and Methods

### 4.1. Chemicals

A green semi-synthetic procedure was carried out to purify oleacein from Coratina cultivar olive leaves of Olea Europaea L. as reported; in detail, oleacein was directly extracted from a water solution of oleuropein in the presence of NaCl under microwave assistance at 180 °C; the crude extract was purified by flash chromatography on silica gel (eluent mixture: CHCl_3_/MeOH 95:5 v/v) [49]. The purity was determined by RP-HPLC, HRMS-ESI, ^1^H-and ^13^C-NMR, as previously reported [11]. Bortezomib and carfilzomib were purchased from Selleckchem (Houston, TX, USA) as DMSO stock-solutions.

### 4.2. Cell Cultures

MM cell lines NCI-H929, RPMI-8226, U266, MM1s and JJN3 were purchased from DSMZ, which certified authentication performed by short tandem repeat DNA typing; the bone marrow stromal cell line HS-5 was purchased from the American Type Culture Collection (Rockville, MD, USA); AMO-1 and AMO-BZB cells were kindly provided by Dr. C. Driessen (University of Tubingen, Tubingen, Germany). The most relevant characteristics of the MM cell lines used are reported in Supplementary Table S1. All these cell lines were immediately frozen and used from the original stock within 6 months. Human MM cell lines were cultured in RPMI-1640 media containing 10% FBS, 2 μmol/L glutamine, 100 U/mL penicillin, and 100 μg/mL streptomycin (GIBCO; Life Technologies, Carlsbad, CA, USA) and tested for mycoplasma contamination. Peripheral blood mononuclear cells (PBMCs) and CD138^+^ cells from BM of MM patients were isolated by Ficoll-hypaque (Lonza Group, Basel, Switzerland), followed by anti-CD138 microbeads (Miltenyi Biotec, Bergish Gladbach, Germany) selection, in accordance with the Declaration of Helsinki following informed consent and Institutional Review Board (University of Catanzaro, Catanzaro, Italy) approval, as previously reported (institutional approval: n.120/2015) [50]; the purity of immunoselected cells was assessed by flow cytometry using a phycoerythrin-conjugated CD138 monoclonal antibody (BD Pharmingen, San Jose, CA, USA; clone DL-101) and was higher than 95%. In co-culture experiments, primary CD138^+^ MM cells (2.5 × 10^5^ cells) were plated in 24-well plates, and left separated from HS-5 stromal cells (2.5 × 10^5^ cells) growing adherent to the plate by a transwell insert of 0.4 μm pore size (Corning, New York, NY, USA).

### 4.3. Cell Viability, Apoptosis and Cell Cycle Assay

Cell viability was evaluated by Cell Titer-Glo (CTG; Promega, Madison, Wisconsin, USA), as previously reported [12]. For colony formation assay, 200 cells were plated in triplicate in 1 mL of mixture composed of 1.1% methylcellulose (MethoCult^TM^ STEMCELL Technologies, Cambridge, UK) in RPMI-1640 + 10% FBS. Crystal violet-stained colonies were scored after 2 weeks under an inverted microscope (Leica DM IL LED, Wetzlar, Germany) at 5× magnification using a grid. Apoptosis was evaluated by flow cytometric (FACS) analysis following Annexin V-7AAD staining (BD Pharmingen, San Jose, CA, USA). Drug interactions were assessed by CalcuSyn 2.0 software (Biosoft, Novosibirsk, Russia), which is based on the Chou-Talalay method. When combination index (CI) = 1, this equation represents the conservation isobologram and indicates additive effects; CI < 1 indicates synergism; CI > 1 indicates antagonism. Cell cycle distribution was evaluated by FACS analysis on MM cells previously treated with oleacein for 24 h, after staining with Propidium Iodide (PI). Cells were collected, washed twice with phosphate-buffered saline (PBS) and fixed in cold 70% ethanol at −20 °C. Before FACS analysis, cells were washed with PBS and stained in 50 μg/mL PI, 100 μg/mL RNase, 0.05% Nonidet P-40 for 1 h at room temperature in the dark. Cell cycle profiles were obtained using Attune NxT Flow Cytometer (Thermo Fisher Scientific, Waltham, MA, USA).

### 4.4. Western Blot and Antibodies

Whole cell protein extracts were prepared using NP40 lysis buffer containing Halt Protease Inhibitor cocktail (Invitrogen, Thermo Scientific, Carlsbad, CA, USA), separated using 4–12% Novex Bis-Tris SDS-acrylamide gels (Invitrogen), and electrotransferred on nitrocellulose membranes (Bio-Rad, Hercules, CA, USA), as described [39]. Then, nitrocellulose membranes were blocked with milk and probed over-night with primary antibodies at 4 °C; then membranes were washed three times in PBS-Tween and incubated with a secondary antibody conjugated with horseradish peroxidase for 2 h at room temperature. Chemiluminescence was detected using SuperSignal West Pico PLUS Chemiluminescent Substrate (Thermo Scientific). Western blot (WB) was performed using Cell Signaling antibodies: PARP (#9532), -Caspase-8 (#9746), -Caspase-3 (#9665), AcH3-Lysin 8 (K9) (#9649P), SP1 (#9389S), HDAC1 (#5356T), HDAC2 (#5113P), HDAC3 (3949P), HDAC4 (#7628S), HDAC6 (#7558P), histone H4 (#2935), histone H3 (#4499), α-tubulin (#2125). Ac-α tubulin (sc-23950), Ac-H4 Ser1/Lys 5/Lys8/Lys Lys 12 (sc-34263), Actin (sc-1616) and -GAPDH (sc-25778) were from Santa Cruz Biotechnology (Dallas, TX, USA); Dnmt1 (ab 13537), Dnmt3a (ab 13888) and Dnmt3b (ab 2851) were from abcam (Cambridge, UK). Densitometric analysis of blots was performed by LI-COR Image Studio Digits Ver 5.0 (Bad Homburg, Germany), expressed as a relative protein unit after normalization with appropriate housekeeping, and reported under each blot. Whole blots of all experiments presented in this study are reported as Supplementary Figures S5–S9.

### 4.5. Reverse Transcription and Quantitative Real Time PCR (qRT-PCR)

Total RNA was extracted from cells using TRIzol^®^ reagent (Gibco, Life Technologies, Carlsbad, CA, USA), following the manufacturer’s instructions. The RNA quantity and quality were assessed through NanoDrop^®^ ND-1000 Spectrophotometer (Waltham, MA, USA). To evaluate transcript changes, 1000 ng of total RNA was reverse-transcribed to cDNA using the “High Capacity cDNA Reverse Transcription Kit” (Applied Biosystems, Carlsbad, CA, USA). The following single-tube TaqMan assays (Applied Biosystems, Carlsbad, CA, USA) were used to detect and quantify genes using the Viia7 DX real time PCR instrument (Life Technologies, Waltham, MA, USA): DNMT1 (Hs00154749_m1), DNMT3a (Hs01027166_m1), DNMT3b (Hs00171876_m1), HDAC1 (Hs02621185_s1), HDAC2 (Hs00231032_m1), HDAC3 (Hs00187320_m1), HDAC4 (Hs01041638_m1), HDAC6 (Hs00195869_m1), and GAPDH (Hs02786624 g1). miRNA expression levels were determined by TaqMan RT-PCR, using the single-tube TaqMan miRNA assays (hsa-miR-29b, assay ID 000413; hsa-miR-22, assay ID 000398, Applied Biosystems) to quantify mature miRNAs, by the use of the StepOne Thermocycler (Thermo Fisher Scientific, Waltham, MA, USA) and the sequence detection system, as previously reported [51]; miRNAs expression levels were normalized on RNU44 (assay ID 001094). Comparative real-time polymerase chain reaction (RT-PCR) was performed in triplicate.

### 4.6. HDAC Activity Assay

Nuclear extracts prepared using NE-PER Nuclear and Cytoplasmic Extraction Reagents kit (Thermo Scientific, catalog #78833) were mixed with Oleacein or DMSO, and then HDAC activity was determined according to the manufacturer’s instructions (BioVision, Zurich, Switzerland; catalog #K331-100). Trichostatin A (TSA) and SAHA were used as positive controls.

### 4.7. Quantification of Global 5-Methylcytosine Levels

Global DNA methylation levels were determined by using 5-mC DNA ELISA kit (Zymo Research, Irvine, CA, USA) as described [52]. Briefly, 100ng of genomic DNA, brought to final volume to 100 µL with 5-mC coating buffer, was denatured at 98 °C for 5min, put in ice for 10’ and then coated on the surface of the ELISA plate wells. After incubation at 37 °C for 1 h, the wells were washed thrice with 200 μL of 5-mC ELISA buffer and then incubated at 37 °C for 1 h with an antibody mix consisting of anti-5-mC (1:2000) and secondary (1:1000) antibodies. Then, the antibody mix was removed from the wells through three consecutive washes with 200μL of 5-mC ELISA buffer. One-hundred microliters of HRP developer was added to each well and incubated at room temperature for 1 h. Absorbance at 405nm was measured using an ELISA plate reader. The percentage of 5-mC was calculated using the second-order regression equation of the standard curve that was constructed by using mixtures of the fully unmethylated and methylated control DNAs, provided by the manufacturer, to generate standards of known 5-mC percentage (0, 5, 10, 25, 50, 75 and 100%).

### 4.8. Statistical Analysis

Each experiment was performed at least three times, and values were reported as mean ± standard deviation. Data were analyzed using Student’s t tests for two group comparisons or a one-way analysis of variance (ANOVA) for multiple comparisons using the Graphpad software (GraphPad Software, La Jolla, CA, USA). *p*-value < 0.05 was considered significant.

## 5. Conclusions

Our results indicate that oleacein, the most abundant EVOO secoiridoid, elicits significant anti-tumor activity by promoting cell cycle arrest and apoptosis, either as a single agent or in combination with the proteasome inhibitor carfilzomib. Moreover, our data highlight an epigenetic impact of oleacein in MM, as demonstrated by the impairment of the MM acetylome, likely *via* Sp1-dependent transcriptional inhibition of HDACs. Altogether, these findings provide the molecular rationale for potential epi-therapeutic anti-MM strategies based on natural agents.

## Supplementary Materials

The following are available online at https://www.mdpi.com/2072-6694/11/7/990/s1, Figure S1: WB of pro-caspase 7, cleaved caspase 7, pro-caspase 9 and cleaved caspase 9 in NCI-H929 cells after 24 h of oleacein treatment, Figure S2: HDAC activity was determined in JJN3 cells treated with oleacein, as reported in materials and methods; TSA was used as positive control. Results are expressed as percentage of HDAC activity as compared to DMSO-treated cells, Figure S3: miR-29b and miR-22 expression levels were determined by qRT-PCR in JJN3 cells treated for 24 h with oleacein; miRNA expression was normalized on RNU44, Figure S4: CTG assay was performed on NCI-H929 cells treated with oleacein (2.5, 5.0 or 10.0 µM) and bortezomib (1.0, 2.0 and 5.0 nM). Results are expressed as percentage of the viability of vehicle-treated cells. The right panel reports values of fraction affected (Fa) and combination indexes (CI) in a triplicate experiment, as calculated by the Calcusyn software, Figure S5: whole blots for Figure 2, Figure S6: whole blots for Figure 3, Figure S7: whole blots for Figure 4, Figure S8: whole blots for Figure 5, Figure S9: whole blots for Figure S1. Table S1: Characteristics of the MM cell lines used in this study.
