# Supplementary material for: Anti-tumor Activity and Epigenetic Impact of the Polyphenol Oleacein in Multiple Myeloma
Source: Cancers (Basel). 2019 Jul 16;11(7):990. doi: 10.3390/cancers11070990 (PMC6679356; doi:10.3390/cancers11070990)
Supplement: Supplementary file 1 [file cancers-11-00990-s001.pdf]

Supplementary Materials: Anti-tumor Activity and Epigenetic Impact of the Polyphenol Oleacein in Multiple Myeloma

Giada Juli, Manuela Oliverio, Dina Bellizzi, Maria Eugenia Gallo Cantafio, Katia Grillone, Giuseppe Passarino, Carmela Colica, Monica Nardi, Marco Rossi, Antonio Procopio, Pierosandro Tagliaferri, Pierfrancesco Tassone and Nicola Amodio

Table S1. Characteristics of MM cell lines.

MM Cell Line	Cytogenetic	*TP53* Status*
AMO-1	t(12;14)	WT
AMO-bzb	t(12;14)	WT
OPM2	t(4;14)	HD
MM1s	t(14;16)	WT
NCI-H929	t(4;14)	WT
RPMI-8226	t(14;16)	HD
JJN3	t(14;16)	HD
U266	t(11;14)	HD

*WT indicates wild type; HD indicates homozygous mutation or homozygous deletion; MM indicates multiple myeloma.

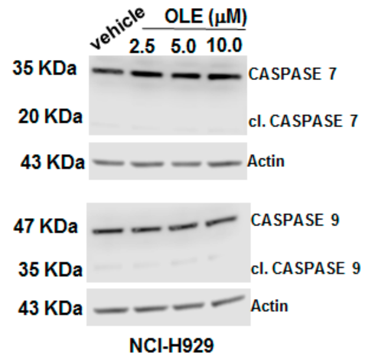

Figure S1. Western Blot (WB) of pro-caspase 7, cleaved caspase 7, pro-caspase 9 and cleaved caspase 9 in NCI-H929 cells after 24 hours of oleacein treatment.

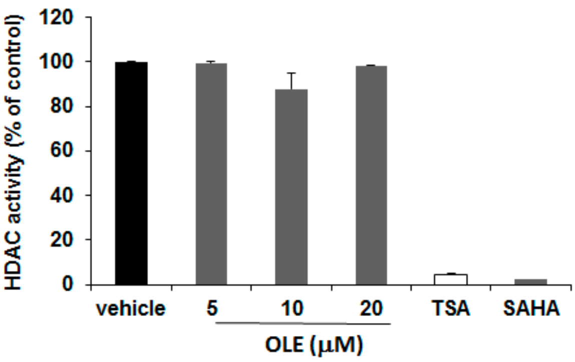

Figure S2. Histone deacetylase (HDAC) activity was determined in JJN3 cells treated with oleacein, as reported in materials and methods; Trichostatin A (TSA) was used as positive control. Results are expressed as % of HDAC activity as compared to DMSO-treated cells. * $p < 0.05$ as compared to vehicle.

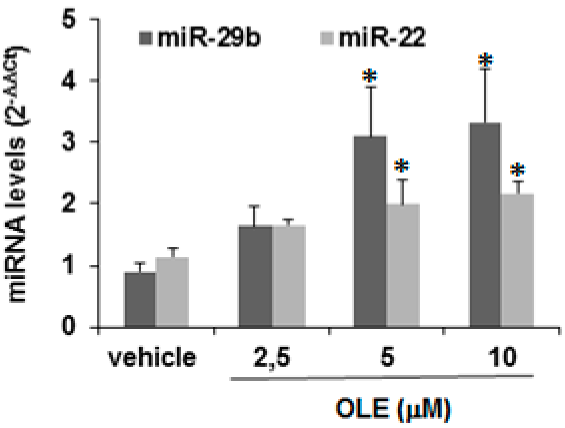

Figure S3. miR-29b and miR-22 expression levels were determined by qRT-PCR in JJN3 cells treated for 24 hours with oleacein; miRNA expression was normalized on RNU44. * $p < 0.05$ as compared to vehicle.

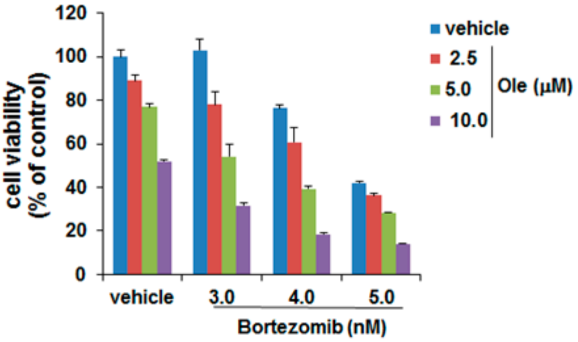

OLE	bortezomib	Fa	CI
2,5	3	0,219	1,23
2,5	4	0,391	1,18
2,5	5	0,637	1,18
5	3	0,458	1,15
5	4	0,606	1,16
5	5	0,716	1,24
10	3	0,683	1,14
10	4	0,814	1,1
10	5	0,859	1,2

Figure S4. Cell Titer Glo (CTG) assay was performed on NCI-H929 cells treated with oleacein (2.5, 5.0 or 10.0 μM) and bortezomib (1.0, 2.0 and 5.0 nM). Results are expressed as the percentage of the viability of vehicle-treated cells. The right panel reports values of fraction affected (Fa) and combination indexes (CI) for each drug combination, as calculated by the Calcusyn software, in a triplicate experiment.

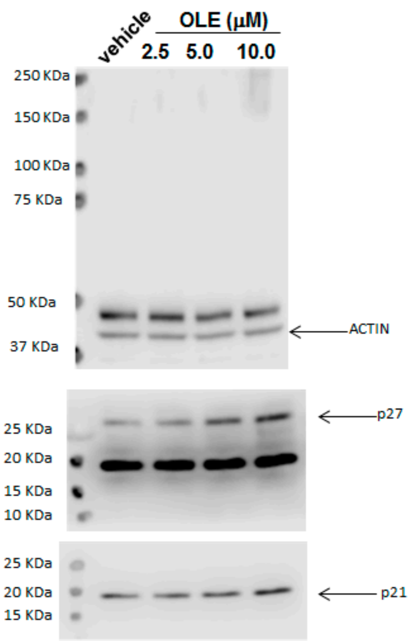

NCI-H929

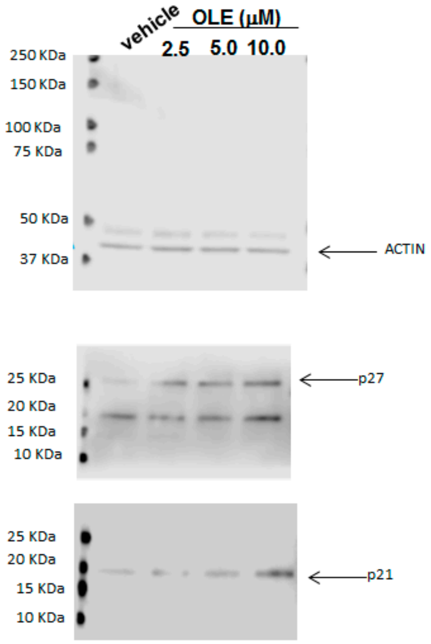

JJN3

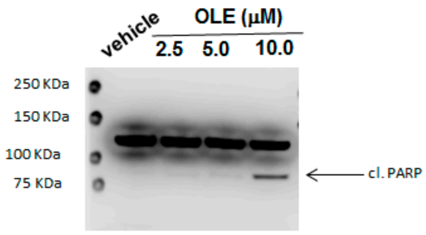

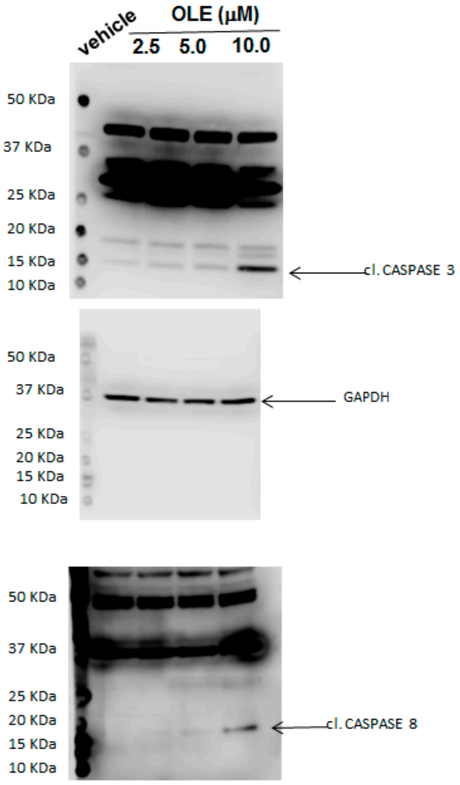

NCI-H929

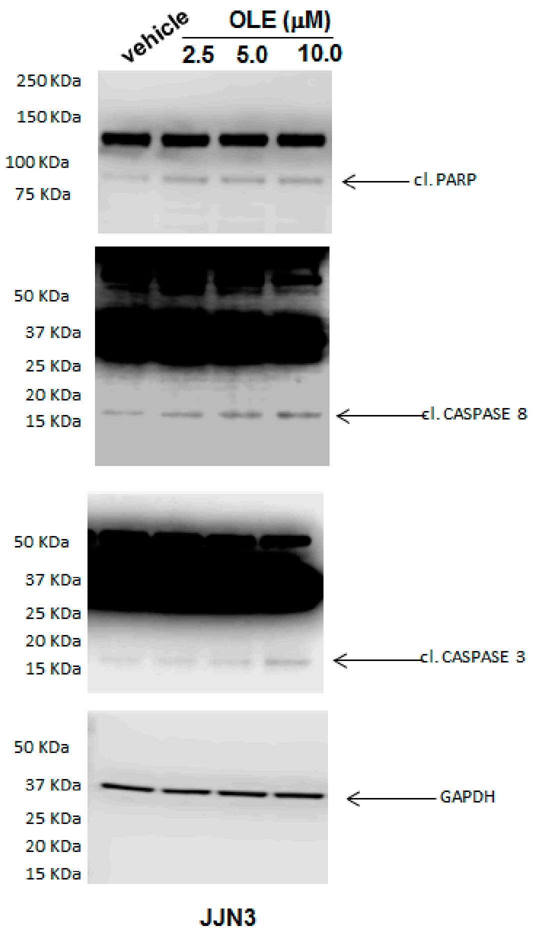

Figure S5. Whole blots for Figure 2.

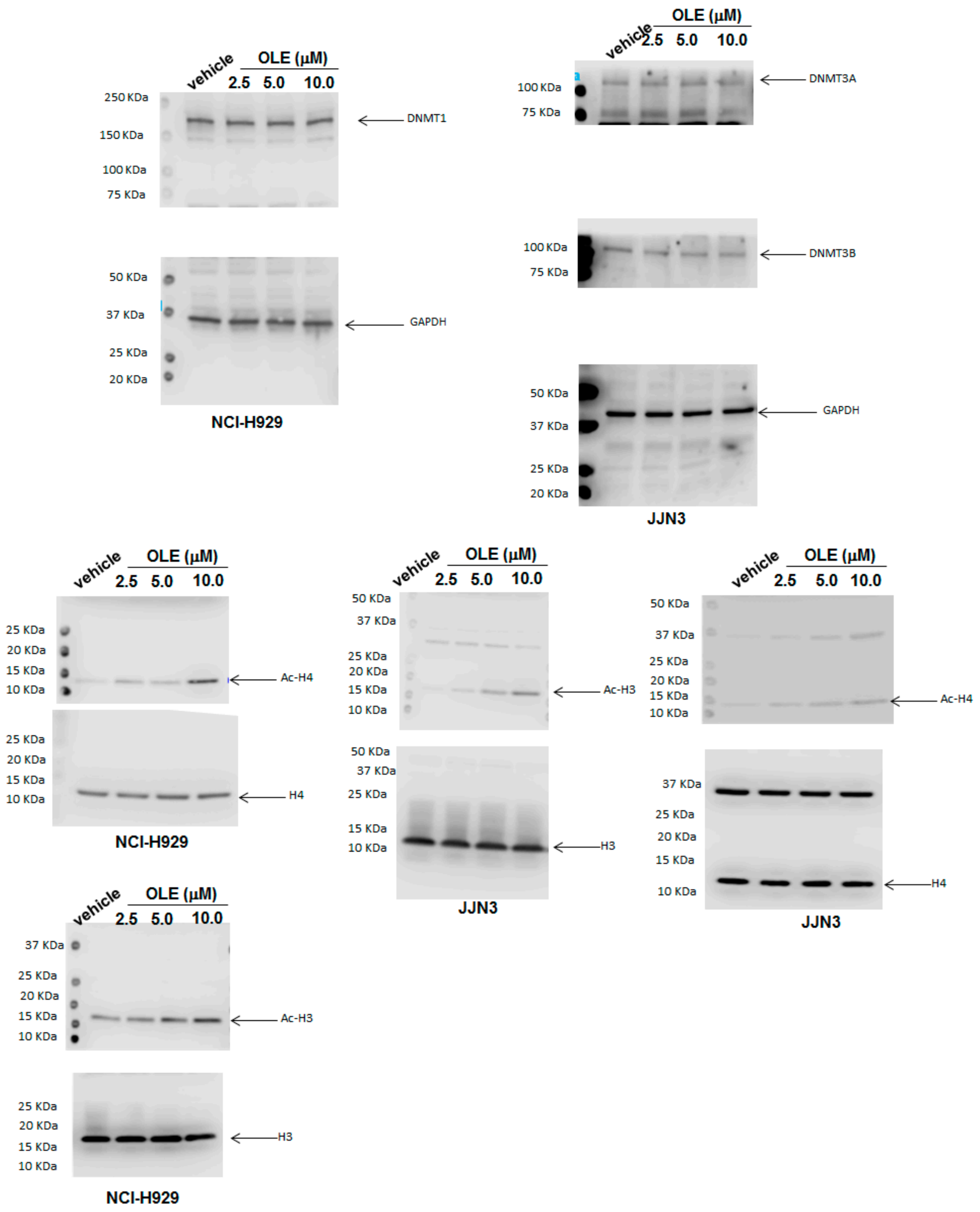
vehicle
OLE (μM)
2.5 5.0 10.0
250 KDa
150 KDa
100 KDa
75 KDa
DNMT1
50 KDa
37 KDa
25 KDa
20 KDa
GAPDH
NCI-H929
DNMT3A
DNMT3B
JJN3
15 KDa
10 KDa
Ac-H4
H4
Ac-H3
H3

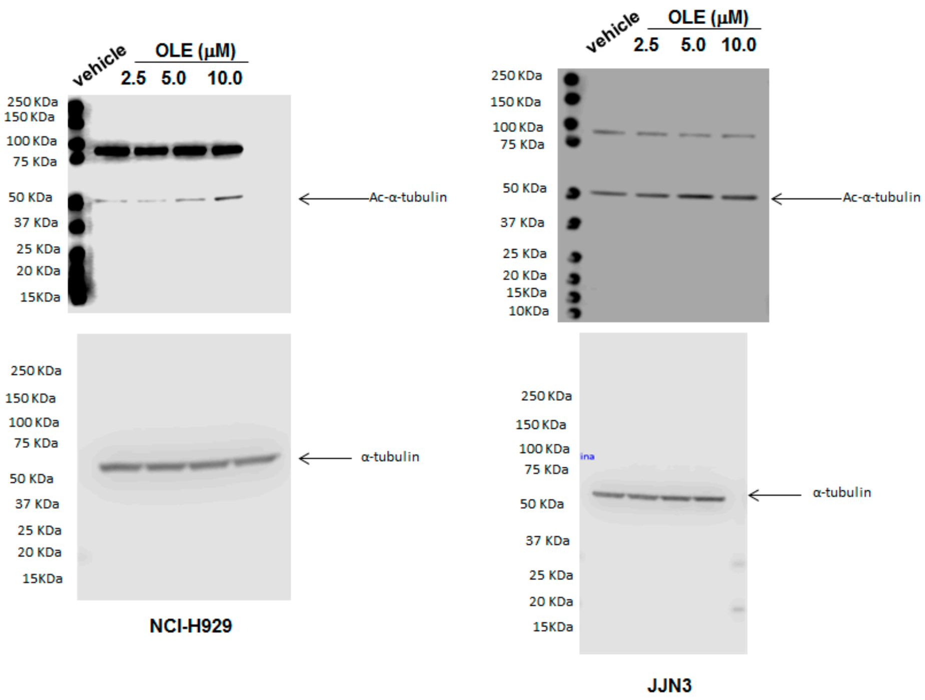

Figure S6. Whole blots for Figure 3.

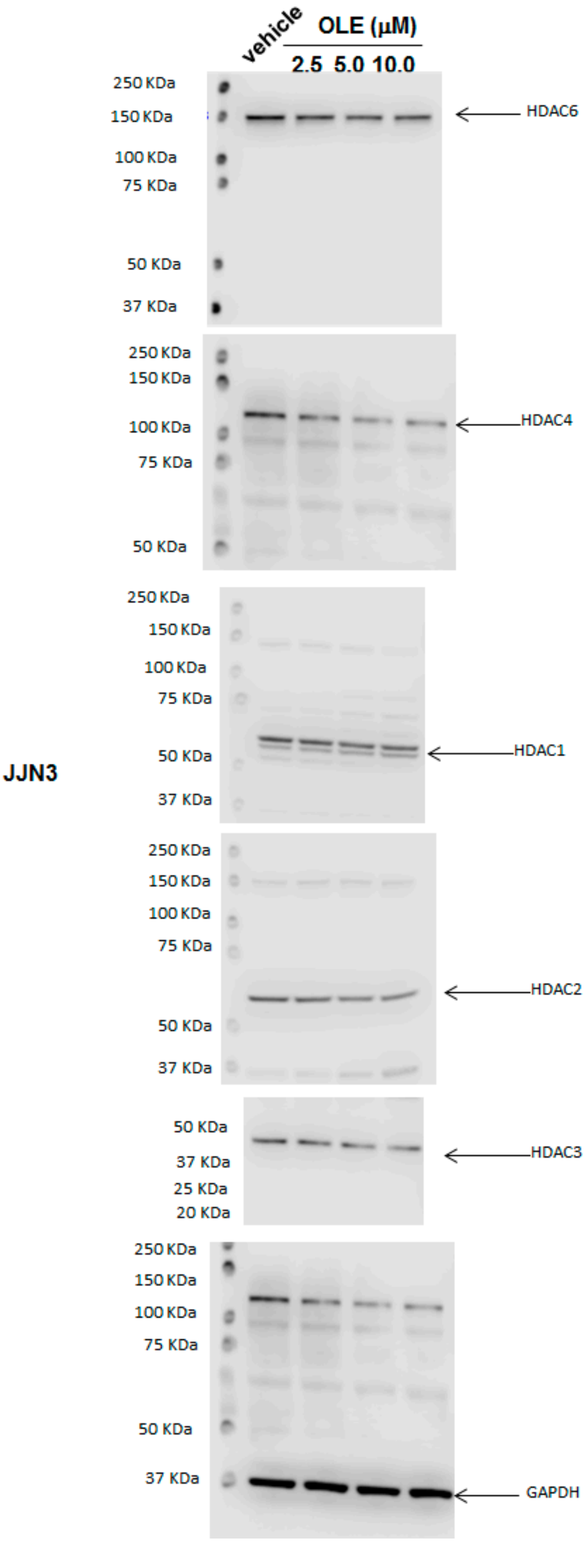
vehicle
OLE (μM)
2.5 5.0 10.0
250 KDa
150 KDa
100 KDa
75 KDa
50 KDa
37 KDa
HDAC6
HDAC4
HDAC1
JJN3
HDAC2
HDAC3
25 KDa
20 KDa
GAPDH

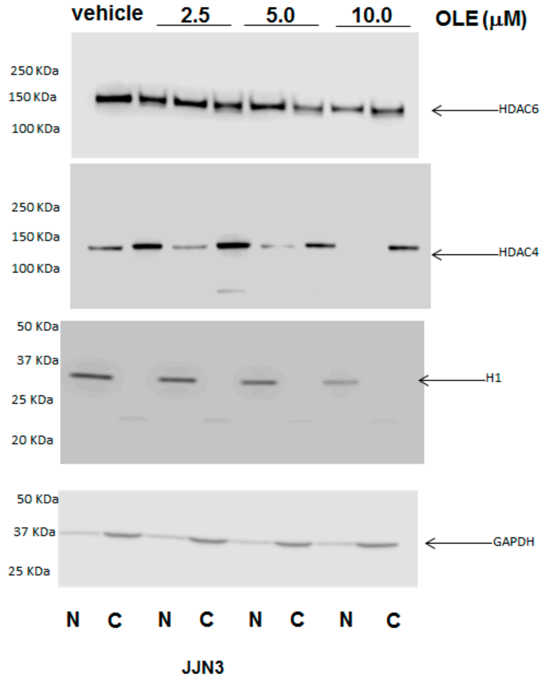
vehicle
2.5
5.0
10.0
OLE (μM)
250 KDa
150 KDa
100 KDa
HDAC6
250 KDa
150 KDa
100 KDa
HDAC4
50 KDa
37 KDa
25 KDa
20 KDa
H1
50 KDa
37 KDa
25 KDa
GAPDH
N C N C N C N C
JJN3

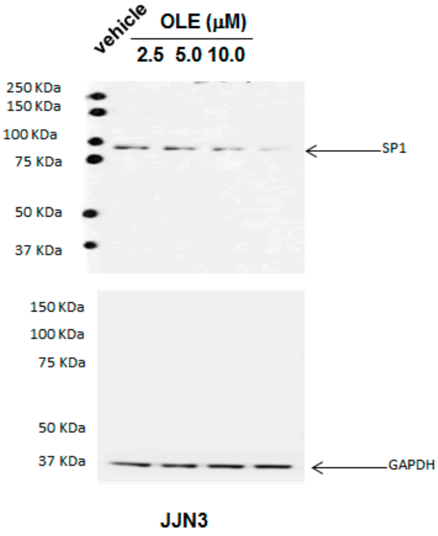
vehicle
OLE (μM)
2.5 5.0 10.0
250 KDa
150 KDa
100 KDa
75 KDa
50 KDa
37 KDa
SP1
150 KDa
100 KDa
75 KDa
50 KDa
37 KDa
GAPDH
JJN3

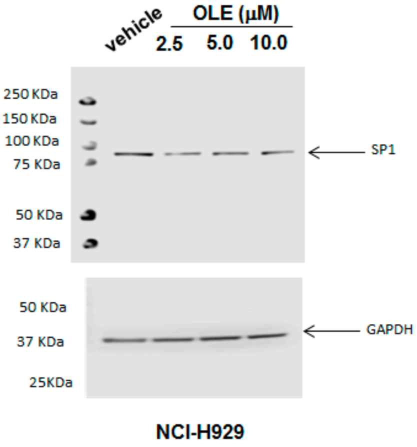
vehicle
OLE (μM)
2.5 5.0 10.0
250 KDa
150 KDa
100 KDa
75 KDa
50 KDa
37 KDa
SP1
50 KDa
37 KDa
25KDa
GAPDH
NCI-H929

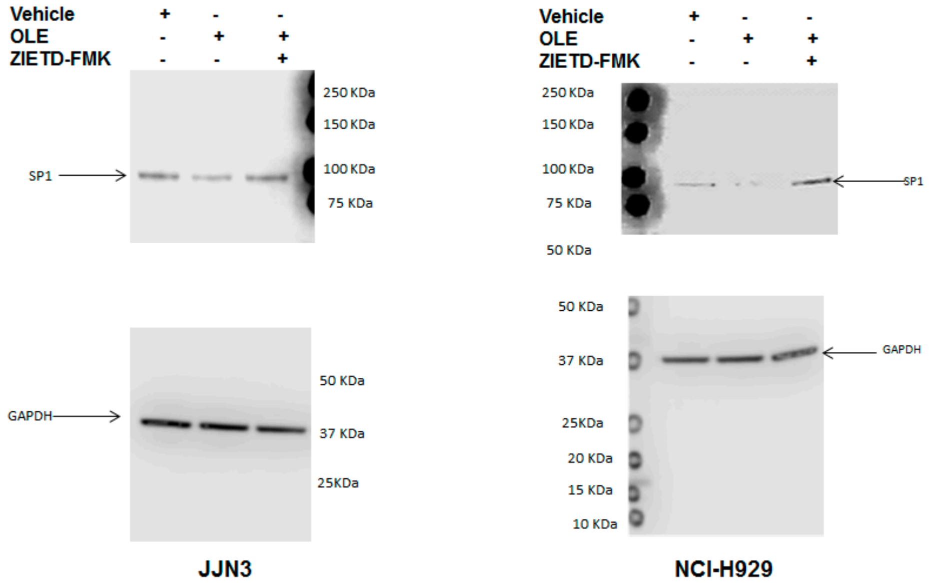

Figure S7. Whole blots for Figure 4.

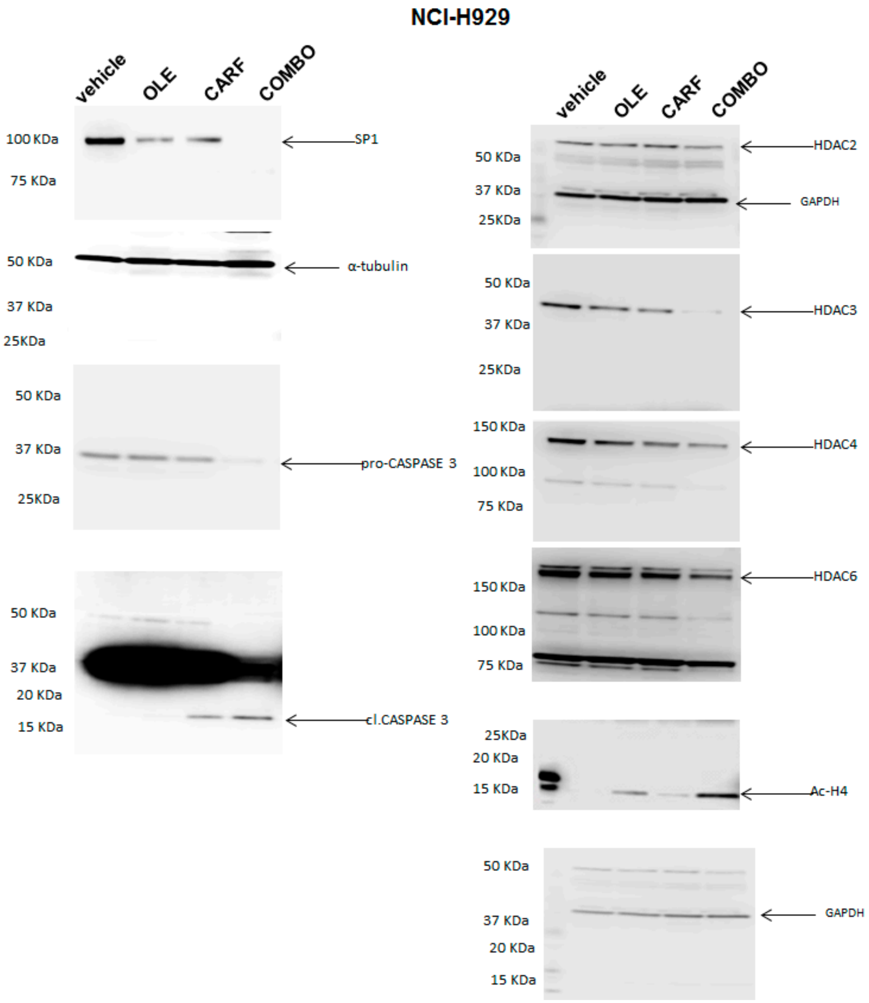

Figure S8. Whole blots for Figure 5.

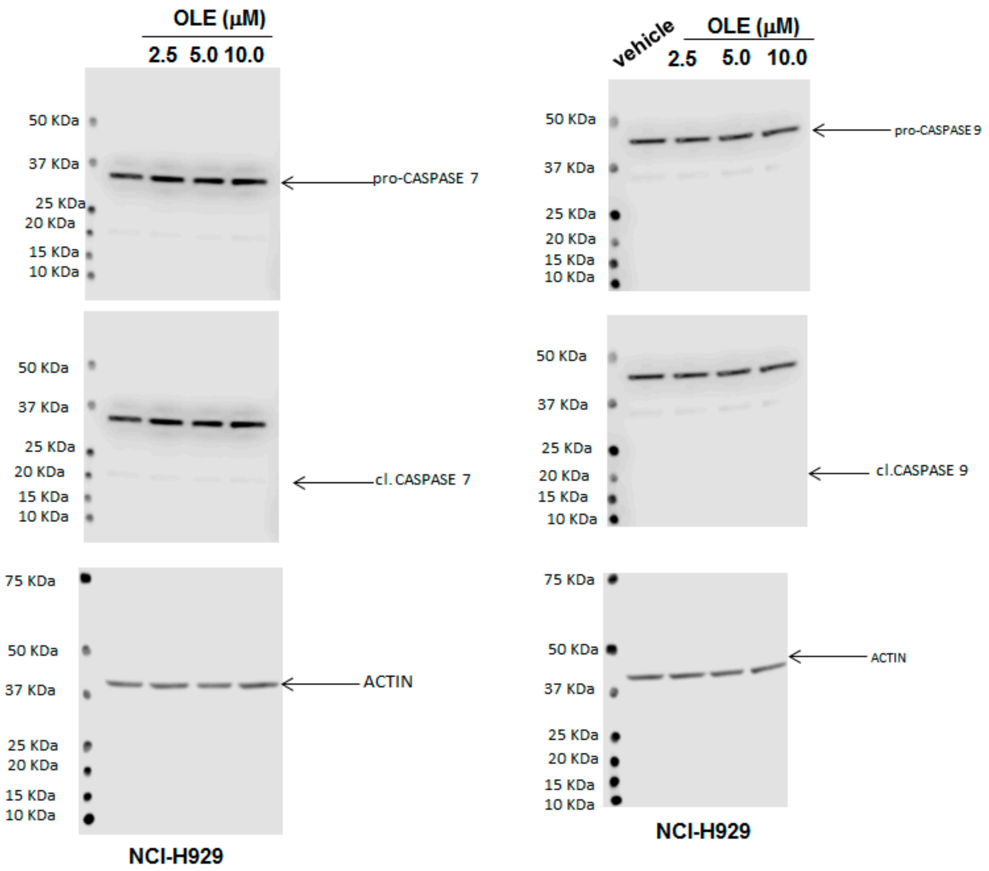

Figure S9. Whole blots for Figure S1.

© 2019 by the authors. Licensee MDPI, Basel, Switzerland. This article is an open access article distributed under the terms and conditions of the Creative Commons Attribution (CC BY) license (http://creativecommons.org/licenses/by/4.0/).
